# The performance evaluation of the state-of-the-art EEG-based seizure prediction models

**DOI:** 10.3389/fneur.2022.1016224

**Published:** 2022-11-24

**Authors:** Zhe Ren, Xiong Han, Bin Wang

**Affiliations:** ^1^Department of Neurology, Zhengzhou University People's Hospital, Zhengzhou, China; ^2^Department of Neurology, Henan Provincial People's Hospital, Zhengzhou, China

**Keywords:** epilepsy, seizure prediction model, EEG, artificial intelligence, seizure occurrence period, seizure prediction horizon, post-processing

## Abstract

The recurrent and unpredictable nature of seizures can lead to unintentional injuries and even death. The rapid development of electroencephalogram (EEG) and Artificial Intelligence (AI) technologies has made it possible to predict seizures in real-time through brain-machine interfaces (BCI), allowing advanced intervention. To date, there is still much room for improvement in predictive seizure models constructed by EEG using machine learning (ML) and deep learning (DL). But, the most critical issue is how to improve the performance and generalization of the model, which involves some confusing conceptual and methodological issues. This review focuses on analyzing several factors affecting the performance of seizure prediction models, focusing on the aspects of post-processing, seizure occurrence period (SOP), seizure prediction horizon (SPH), and algorithms. Furthermore, this study presents some new directions and suggestions for building high-performance prediction models in the future. We aimed to clarify the concept for future research in related fields and improve the performance of prediction models to provide a theoretical basis for future applications of wearable seizure detection devices.

## Introduction

**Figure 1:**
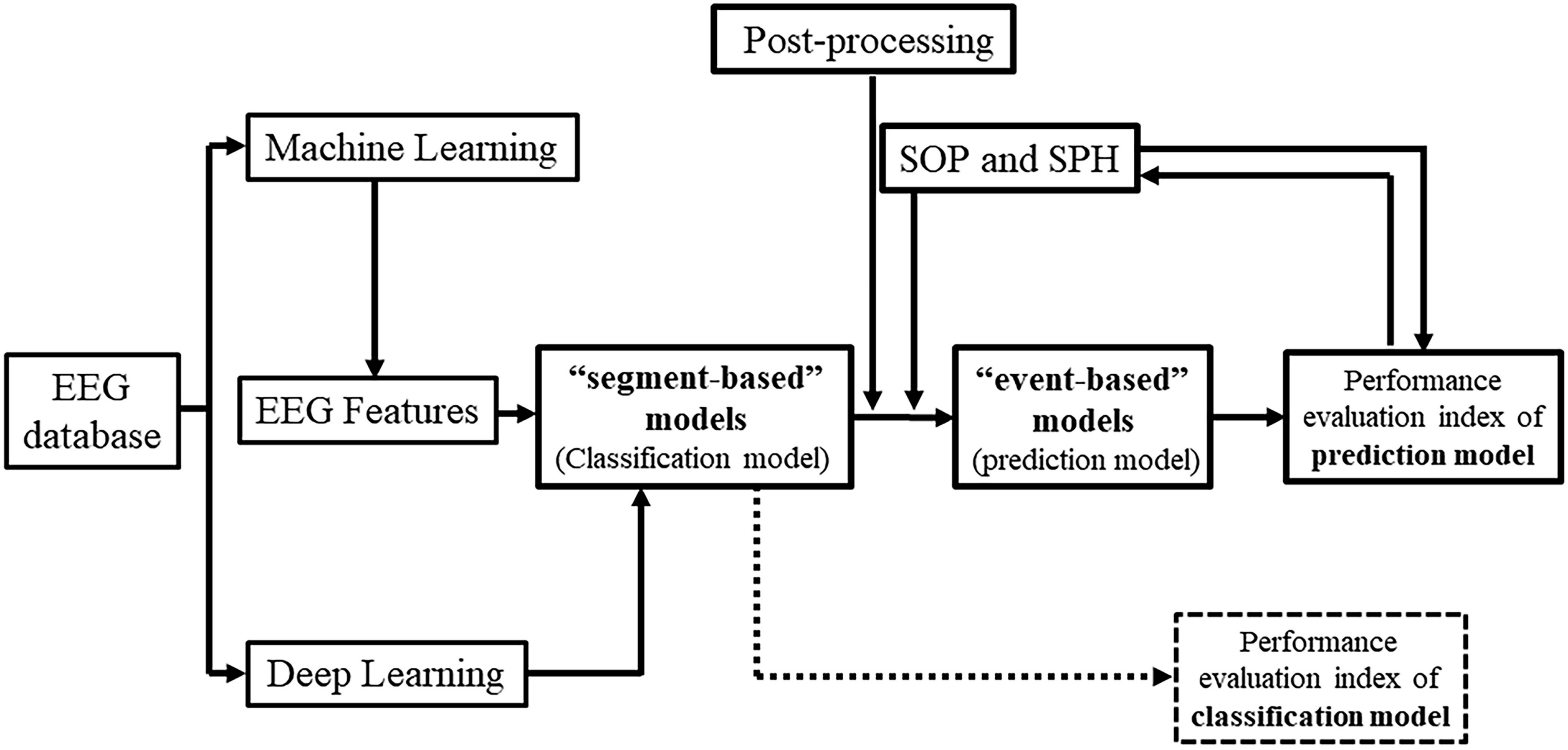
Flowchart for based-on-EEG seizure prediction.

Epilepsy is one of the most common chronic diseases of the nervous system, affecting approximately 70 million people worldwide with a prevalence rate of 4.0–7.0‰ (1). Notably, about 30–40% of epilepsy patients exhibit treatment resistance to drug therapy (2) and suffer from complex epilepsy symptoms, called drug-refractory epilepsy (DRE). Even epilepsy patients who show a positive response to antiepileptic drugs initially may face the risk of recurring episodes of epileptic seizures at any time, causing excessive stress, anxiety, and deviation from their normal lifestyle (3). In clinics, as well as in scientific investigations, electroencephalogram (EEG) is an important tool for the diagnosis of epilepsy. Since the first EEG-based publications by Iasemidis and Sackellares group in the 1980's and 1990's, that provided evidence of seizures being non-random events and hence predictable (4–17), the first prospective seizure prediction algorithm running real-time on continuous EEG data was developed by Iasemidis et al. (18). Non-linear features of the EEG, such as the largest Lyapunov exponent and phase changes in the state space, were extracted over time to identify dynamical spatial entrainment changes in the preictal period between critical brain sites. The algorithm across patients reached average values of sensitivity of 84%, false prediction rate of 0.12/h, and prediction time prior to seizures (early warning time) of 74.4 min. This first seizure prediction algorithm was automated and adaptive, the precursor of the current so-called event-based models, needed to detect the occurrence of the first seizure for initialization of its parameters per patient and did not need any predetermined (i.e., arbitrary chosen) preictal (and hence neither SOP nor SPH) period. Seizure prediction became a whole new field in brain research thereafter, and a hot topic due to its many potential applications in the diagnosis, prognosis and treatment of epilepsy and potentially of other brain dynamical disorders (3, 19–21). With the development and application of Artificial Intelligence (AI) technology for clinical and diagnostic purposes, the use of Machine Learning (ML) and Deep Learning (DL) in constructing models for seizure prediction based on EEG features has become a popular method in epilepsy management (Figure 1) (16–18, 22, 23).

Remarkable progress has been made in the field of seizure detection by EEG using AI techniques, and at least three instruments that have been clinically tested received well acceptance (24, 25). However, there are still some difficulties in predicting seizures precisely. By reviewing the related literature and the depth of research, we found that one of the reasons is the existing gap between the research outcomes and their clinical application since most researchers develop high-performance prediction models without considering the real-life parameters. For example, some models do not use post-processing techniques, leading to the possibility that multiple warning messages will be appearing before a single seizure which, if applied in practice, would seriously disrupt patients' lives. Seizure Occurrence Period (SOP) and Seizure Prediction Horizon (SPH) settings are not often considered in this practical sense, which can lead to excessive anxiety and seriously affect the patient's daily activities. Moreover, as presented in previous studies, evaluation metrics of “event-based” and “segment-based” model performance may sometimes seem confusing.

Kuhlmann et al. (26) provided a detailed overview of the field of seizure prediction, indicating its future directions. Others have outlined particular areas from previous studies, such as features, methods (21, 27–29), model selection, and Brain-Computer Interfaces (BCI) (3, 20, 30, 31), etc. However, there are barely any review studies specifically focusing on the AI-guided construction of “event-based” models for predicting seizures from EEG findings, and the importance of post-processing techniques, SOP and SPH. This study aimed to clearly demonstrate the relationship between “segment-based” and “event-based” prediction models, and summarize the underlying factors that affect these prediction models. Moreover, we highlight the importance of post-processing techniques and their impact on future BCI applications.

## Segment-based and event-based models for predicting seizures

**Figure 2:**
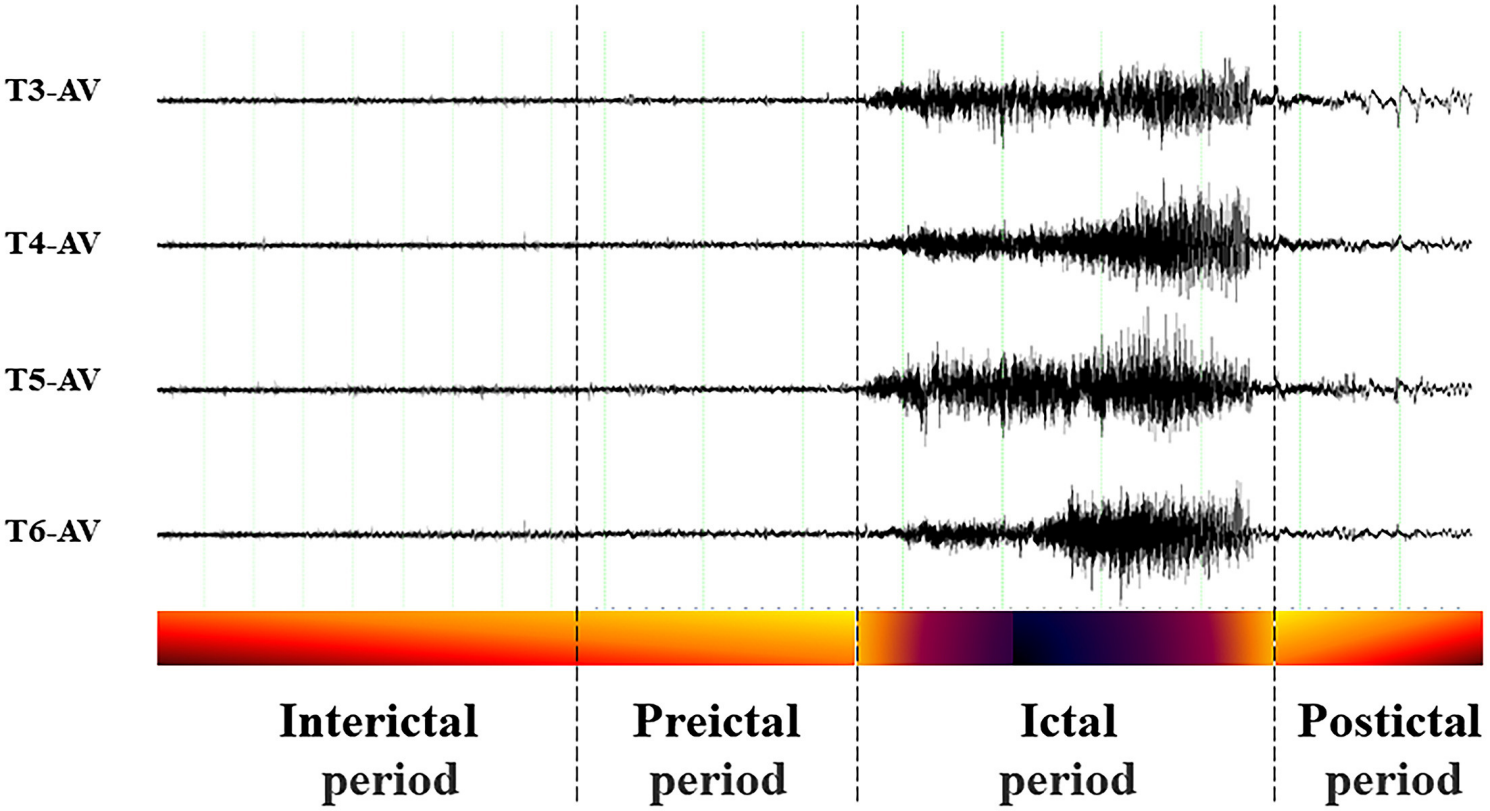
Example of perictal EEG and the respective periods. Interictal period: away from seizures; Preictal period: immediate before a seizure; *Ictal period*: spans the seizure itself; Postictal period: immediate after a seizure.

The EEG recordings of epilepsy patients can be divided into four distinct periods (Figure 2): the ictal period that spans the duration of a seizure; the preictal period, that is, the to be detected (but in seizure prediction models it is specified) period immediate prior to a seizure's onset where signs of the upcoming seizure may exist; the postictal period, that is, the to be detected (but in seizure prediction models it is specified) period immediate after seizure's end and up to the interictal period; and the interictal period, that is, the seizure-free (baseline) period between seizures, that excludes the preictal and postictal periods, and in which epilepsy patients remain most of the time. Thus, the essence of seizure prediction is to identify the beginning of the preictal period as early as possible (minutes to hours) before a seizure's occurrence and issue warnings for the impending seizure. (In the cases that the preictal period is found to last days, its detection is more a subject of the field of seizure susceptibility than of seizure prediction).

By dividing interictal and preictal EEG data into windows of the same size, a classification model, called the “segment-based” prediction model, can be constructed to detect the predetermined preictal EEG period (= SOP + SPH) prior to seizures (same length of preictal period is typically assumed before all seizures) (32, 33). This model is designed to accurately distinguish between predetermined preictal and interictal EEG segments, and if the model becomes more accurate in identifying the number of preictal segments, it demonstrates that it could in practice identify preictal changes with high performance (necessary condition). It is not sufficient to construct a classification model that identifies preictal segments; it is meaningless to alert in practice with a real-time warning every time a segment is identified as preictal. Also, there will inevitably be errors in the classification of preictal windows, which will most likely result in too frequent warnings and a large number of false alarms. Thus, the results of the segment-based model need to be post-processed in order to be able to realistically apply its predictions in the clinic.

When the occurrence of a seizure event is predicted and a warning sign is issued as soon as the model identifies the first preictal window, or after the post-processing of the results on the windows from the preceding segment-based analysis by techniques known as output regularization (33–36), it is called “event-based” seizure prediction model (33, 34, 37). Hence, this model can also be used for seizure detection, where a seizure is expected to occur after the pre-determined preictal period of the model (38–40).

The performance of the event-based and segment-based prediction models is a decisive factor in deciding whether to use them in clinical practice, as only high-performance prediction models could accurately predict seizures without a significantly large number of false alarms affecting patients' psychological status.

## Metrics and parameters of seizure prediction models' performance

### Evaluation metrics of performance

Some of the common metrics used to evaluate the performance of a seizure prediction model include sensitivity (Sen), specificity (Spe), accuracy (Acc), false prediction rates per hour (FPR/h), warning time, warning time ratio (41, 42). The performance evaluation metrics of “segment-based” and “event-based” prediction models are different in their meaning as the models themselves are based on different assumptions. Table 1 describes the relationship and conceptual issues regarding evaluation metrics between the “segment-based” and “event-based” prediction models (68–70). Table 2 highlights the evaluation metrics of the performance of the “event-based” prediction models. In most of the “event-based” prediction models, the performance is evaluated primarily by the sensitivity and FPR/h metrics (22, 42, 68). Table 3 highlights the evaluation metrics of the performance of the “segment-based” prediction models.

**Table 1:** Evaluation metrics for “segment-based” and “event-based” models for predicting seizures.

**“segment-based” models** (Classification model) (22, 33, 34, 40, 43–52)	*TP (True Positive):* The number of preictal windows correctly identified by the classifier as preictal. *FP (False Positive):* The number of interictal windows incorrectly identified as preictal by the classifier. *TN (True Negative):* The number of interictal windows correctly identified as interictal by the classifier. *FN (False Negative):* The number of preictal windows incorrectly identified by the classifier as interictal.
Sensitivity, SEN Also known as recall	*Meaning:* Ability of the preictal window to be correctly identified by the classifier. SEN = TP/(TP + FN) *Significance:* It measures the ability of the classification model to identify the preictal period and is proportionally related to the model performance.
Specificity, Spe	*Meaning:* the ability of the interictal window to be correctly identified by the classifier. Spe = TN/(TN + FP) *Significance:* It measures the ability of the classification model to identify the interictal period, and the higher it is, the better the model performance is.
Accuracy, Acc	*Meaning:* the ability of the classifier to correctly identify interictal and preictal periods. Acc = (TP + TN)/(TP + TN + FN + FP) *Significance:* The ability of the classification model to identify the interictal and preictal periods is measured, with higher levels demonstrating better model performance.
False prediction rate, FPR	*Meaning:* The proportion of interictal periods incorrectly identified by the classifier as preictal. FPR = FP/(TN + FP) *Significance:* It measures the false recognition layer of the classification model for the interictal period, and the lower it is, the better the model performance is.
**“event-based” models** (prediction model) (18, 22, 23, 33, 40–44, 46, 47, 50, 53–67)	
Sensitivity, Sen Also known as recall	*Meaning:* The proportion of the number of seizures correctly predicted by the prediction model to the total number of seizures in the test set. *Significance:* It measures the prediction performance of the prediction model, and is the most important indicator of the performance of the prediction model; the greater the sensitivity, the better.
Average false prediction Rate per hour, FPR/h	*Meaning:* The average number of false alerts per hour. *Significance:* It measures the prediction performance of the forecasting model, and is an important indicator of the performance of the forecasting model, the lower it is, the better the performance of the forecasting model.
Early warning time	*Meaning:* The distance between the time when the prediction model gives a warning and the time of seizure in the test set. *Significance:* It is an important indication of the performance of the prediction model, the longer the time, the better.

**Table 2:** Previous studies of event-based models for predicting seizures.

**Classifier**	**EEG datasets**	**Predeter mined preictal and window lengths**	**Predeter mined SOP and SPH**	**Evaluation indicators**
CSSPM (68)	CHB-MIT 11 patients	Preictal = 30 min Window = 30 s	SPH = 5 min SOP = 30 min	Sen: 78.5% FPR: 0.44/h
CNN (71)	CHB-MIT 10 patients	Preictal = 30 min Window = 5 s	SPH = 5 min SOP = 30 min	Sen: 99.81% FPR: 0.005/h
STS-HGCN-AL (55)	CHB-MIT 19 patients	Preictal = 15 min Window = 5 Sec	SPH = 1 min SOP = 15 min	Sen: 95.5% FPR: 0.109/h
SDCN (23)	CHB-MIT 22 patients AES 5 dogs, 2 patients Melbourne 15 patients	Preictal = 60 min Window = 30 Sec Preictal = 66 min Window = 30 Sec Preictal = 60 min Window = 30 Sec	SPH = 5 min SOP = 60 min	Sen: 98.9% FPR:0.06/h Sen: 88.45% FPR: NA Sen: 89.52% FPR: NA
FRCNN (72)	Freiburg 20 patients CHB-MIT 16 patients	Preictal = 30 min Window = 1 Sec	SPH = NA SOP = 30 min	Sen: 91% FPR: 0.06/h Sen: 85% FPR: 0.14/h
DNN CNN LSTM (40)	CHB-MIT 15 patients	Preictal = 3 min Window = 1 Sec	SPH = NA SOP = 40 min	Sen: 76.6% FPR: 0.71/h Sen: 90.66% FPR: 0.204/h Sen: 90.72% FPR: 0.241/h
EA (59)	Freiburg 19 patients	Preictal = 40 min Window = 5 Sec Preictal = 50 min Window = 5 Sec Preictal = 60 min Window = 5 Sec	SPH = 10 min SOP = 40.46 min	Sen: 38% FPR:1.03/h Sen: 36% FPR:0.76/h Sen: 37% FPR: 0.58/h
Gradient boosting classifier (73)	Freiburg 20 patients	Preictal = 30 min Window = 4 Sec	SPH = 2 min SOP = 30 min SPH = 2 min SOP = 50 min	Sen: 90.42% FPR: 0.12/h Sen: 91.67% FPR: 0.10/h
CSP and CNN (42)	CHB-MIT 23 patients	Preictal = 30 min Window = 5 Sec	SPH = 0 min SOP = 30 min	Sen: 92.2% FPR: 0.12/h
MLP (53)	CHB-MIT 10 patients	Preictal = 30 min Window = 16 Sec	SPH = 0 min SOP = 120 min	Sen: 89.81% FPR: 0.081/h
CNN and DTF (74)	Freiburg 19 patients	Preictal = 30 min Window = 10 Sec	SPH = 5 min SOP = 30 min	Sen: 90.80% FPR: 0.08/h
FDMR (75)	CHB-MIT 21 patients	Preictal = 60 min Window = 10 Sec	SPH = 0 min SOP = 60 min	Sen: 100% FPR: 0.07/h
DCAE + Bi-LSTM (69)	CHB-MIT 8 patients	Preictal = 60 min Window = 5 Sec	SPH = 0 min SOP = 60 min	Sen: 99.72% FPR: 0.004/h
3D CNN (54)	CHB-MIT 16 patients	Preictal = 30 min Window = 5 Sec	SPH = 1 min SOP = 30 min	Sen: 85.7% FPR: 0.096/h
DTF (60)	Freiburg 21 patients	Preictal = 10 min Window = 5 Sec	SPH = 10 min SOP = 50 min	Sen: 79.76% FPR: 0.33/h
LRCN (33)	sEEG 15 patients	Preictal = 30 min Window = 10 Sec	SPH = 5 min SOP = 30 min	Sen: 100% FPR: 0.04/h
BLDA (41)	Freiburg 21 patients	Preictal = 60 min Window = 4 Sec	SPH = 10 Sec SOP = 30 min SPH = 10 Sec SOP = 50 min	Sen: 85.11% FPR: 0.08/h Sen: 93.62% FPR: 0.08/h
CNN (57)	Freiburg 13 patients CHB-MIT 13 patients AES 5 dogs, 2 patients	Preictal = 60 min Window = 30 Sec	SPH = 5 min SOP = 30 min	Sen: 81.4% FPR: 0.06/h Sen: 81.2% FPR: 0.16/h Sen: 75% FPR: 0.21/h
CNN (56)	MSSM 28 patients CHB-MIT 22 patients	Preictal = 10 min Window = 1 Sec	SPH = 0 min SOP = 10 min	Sen: 87.8% FPR: 0.142/h
LSTM CNN (43)	CHB-MIT 22 patients	Preictal = 120 min Window = 5 Sec	SPH = 0 min SOP = 120 min	Sen: 100% FPR: 0.02/h
SVM (22)	the European Epilepsy Database 216 patients	Preictal = 10–40 min Window = 5 Sec	SPH = 10 Sec SOP = 28 min	Sen: 38.24% FPR: 0.2/h
SI (61)	5 patients iEEG sEEG	Preictal = 10–30 min Window = 30 Sec	SPH = 0 Sec SOP = 10 min	Sen: 84% FPR: 0.79/h Sen: 72% FPR: 1.01/h
Non-linear index (76)	Freiburg 10 patients	Preictal = 50 min Window = 10 Sec	SPH = 10 Sec SOP = 30 min SPH = 10 Sec SOP = 50 min	Sen: 86.7% FPR: 0.126/h Sen:92.9% FPR: 0.096/h
LS-SVM (58)	Freiburg 21 patients	Preictal = 5 min Window = 10 Sec	NA	Sen: 91.95% FPR: 2.14/per
Linear SVM (77)	Freiburg 18 patients CHB-MIT 17 patients	Preictal = 60 min Window = 4 Sec	NA	Sen: 100% FPR: 0.0324/h Sen: 98.68% FPR: 0.0465/h
MLP (78)	CHB-MIT 23 patients	Preictal = 60 min Window = 30 Sec	NA	Sen: 97.27% FPR: 0.00215/h
SVM (79)	The European Epilepsy Database sEEG 16 patients iEEG 8 patients	Preictal = 10–40 min Window = 5 Sec	SPH = NA SOP = 10–40 min	Sen: 73.98% FPR: 0.06/h Sen: 78.36% FPR: 0.15/h
SVM (80)	Freiburg 18 patients	Preictal = 30 min Window = 20 Sec	SPH = NA SOP = 30 min	Sen: 92.5% FPR: 0.2/h
ASPA (18)	iEEG 5 patients	Preictal = 180 min Window = 10.24 Sec	NA	Sen:84% FPR: 0.12/h

CSSPM, consistency-based semi-supervised seizure prediction model; STS-HGCN-AL, spatio-temporal-spectral hierarchical GCN with an active preictal interval learning scheme; SDCN, semi-dilated convolutional network; FRCNN, Fourier ratio convolutional neural network; DNN, deep neural network; CNN, convolutional neural network; LSTM, long short-term memory; EA, evolutionary algorithm; CSP, common spatial pattern; MLP, multilayer perceptron; DTF, directed transfer function; DCAE, deep convolutional autoencoder; Bi-LSTM, bidirectional long short-term memory recurrent neural network; FDMR, frequency-domain model ratio; LRCN, long-term recurrent convolutional network. BLDA, bayesian linear discriminant analysis; SI, similarity index; SVM, support-vector machine; LS-SVM, least square-support vector machine; AES dataset Kaggle, the American epilepsy society seizure prediction challenge dataset; MSSM dataset, the mount sinai epilepsy center at the mount Sinai Hospital; ASPA, adaptive seizure prediction algorithm.

**Table 3:** Previous studies of segment-based models for predicting seizures.

**Classifier**	**EEG datasets**	**Predeter mined preictal and window lengths**	**Evaluation indicators**
SVM + CNN + LSTM (45)	CHB-MIT 22 patients AES 5 dogs, 2 patients	Preictal = 60 min Window = 30 Sec	Sen: 96.28% Spe: 95.65% Sen: 94.20% Spe: 95.81%
CNN + Waxman graph (34)	19 patients iEEG+sEEG	Preictal = 10 min Window = 1 Sec	Acc: 98.2%
RDANet (46)	CHB-MIT 13 patients	Preictal = 30 min Window = 5 Sec	Sen: 89.33% Spe: 93.02% Acc: 92.07% AUC: 91.26%
TASM-ResNet (47)	AES 5 dogs, 2 patients	Preictal = 10 min Window = 30 Sec	Sen: 76.1% Spe: 81% Acc: 80.5% AUC: 89.8%
CNN (48)	SEEG	Preictal = 23.6 min Window = 5 Sec	Acc: 94.1% Sen: 91.8% Spe: 90.5%
ANN (44)	CHB-MIT 22 patients	Preictal = 1000 Sec Window = 500 Sec	Sen: 91.82% Spe: 99.11% Acc: 98.66% AUC: 84%
SST-based CNN (49)	IKCU 16 patients CHB-MIT 22 patients	Preictal = 5 min Window = 5 Sec	Acc: 99.06% Sen: 99.18% Acc: 99.63% Sen: 99.52%
DNN			Sen: 51.83% Spe: 75.29% Acc: 64.55%
CNN	CHB-MIT 15 patients	Preictal = 3 min Window = 5 Sec	Sen: 88.22% Spe: 90.47% Acc: 89.21%
LSTM (40)			Sen: 91.46% Spe: 91.58% Acc: 90.94%
CNN (50)	9 patients iEEG	Preictal = 5 min Window = 30 Sec	Acc: 99.69%
SVM (51)	Freiburg 5 patients	Preictal = 5 min Window = 10 Sec	Sen: 95.2% Spe: 99.4% Acc: 97.42%
LRCN (33)	15 patients sEEG	Preictal = 30 min Window = 10 Sec	Sen: 91.88% Spe: 86.13% Acc: 93.4%
LSTM+CNN (43)	CHB-MIT 22 patients	Preictal = 120 min Window = 5 Sec	Sen: 99.84% Spe: 99.86%
SVM (22)	The European Epilepsy Database 216 patients	Preictal = 10–40 min Window = 5 Sec	Sen: 35.34% Spe: 76.53% Acc: 63.62%
Decision tree (52)	Bonn 5 patients	Preictal = 23.6 Sec Window = 23.6 Sec	Sen: 99% Spe: 99.5% Acc: 95.67%

RDANet, a dual self-attention residual network, TASM-ResNet, time attention-based simulation module—residual network; ANN, artificial neural network; SST, Fourier-based synchro-squeezing transform; IKCU dataset, recorded at Izmir Katip Celebi University School of Medicine, Department of Neurology.

Different ML and DL algorithms can influence the performance of the constructed epilepsy prediction models, with some generally applicable algorithms resulting in more stable performances. In a study (43) of a seizure prediction model combining a Long Short-Term Memory (LSTM) network with convolutional neural networks (CNN), authors used a public dataset of scalp EEG (sEEG) recordings from 23 patients with 185 seizures. The sensitivity of the model in “event-based” prediction was up to 100%, with FPR = 0.02/h and a predetermined preictal window (period) of 2 h. However, the authors also therein state that their approach does not allow to evaluate the prediction time, that is, the duration from the first preictal segment identification to the actual seizure onset. Another study (33) used Long-term Recurrent Convolutional Network (LRCN) to construct a seizure prediction model from EEG data of 15 epilepsy patients and achieved 100% sensitivity and FPR of 0.04/h in the “event-based” model. Zhao et al. (71) reportedly achieved a sensitivity of 99.81% and FPR of 0.05/h in a CNN-based prediction model constructed using sEEG recordings from 10 epilepsy patients in a public dataset.

Although models constructed using these conventional algorithms have shown satisfactory results, it is still difficult to apply them to clinical practice, largely because DL-guided models can easily cause overfitting, resulting in weak generalization when their assumptions (e.g. the predetermined values of their parameters) are not realized. Additionally, there is a need to minimize the computational load (time and power consumption) while maintaining a high level of performance, especially in implantable and wearable devices where such a load may be critical (71). The introduction of new concepts through innovative algorithms and models to enable prospective real-time clinical applications while maintaining the their retrospective high performance levels is the current mainstream approach to developing high-end AI-guided seizure prediction models.

The performance of models constructed by new algorithms and technologies is generally lower than that of models constructed based on matured algorithms, which may be related to the fact that the new algorithms are not yet perfectly optimized for the desired tasks. Bomela et al. (81) exploited “Inferring The Connectivity Of Networks (ICON)”, a new technique for analyzing the connectivity features of functional brain networks, and the final prediction model obtained a sensitivity of 93.62% and FPR of 0.16/h in the public dataset of sEEG data from 17 patients. Recently, another author proposed the Consistency-based Semisupervised Seizure Prediction Model (CSSPM) for epileptic seizure prediction (68), which obtained a mean sensitivity of 78.5% and the FPR of 0.44/h in sEEG recordings from 11 patients in a public dataset. It has been argued that a FPR value above 0.15/h is not acceptable (82) because even though the sensitivity is high, a high FPR would lead to too frequent false alarms. In another study, authors reported a sensitivity of 89.81% and FPR of 0.08/h in sEEG recordings of 10 patients using the Hilbert Vibration Decomposition (HVD) method to extract features and construct a CNN-based seizure prediction model (53).

### Parameters of performance: Seizure occurrence period (SOP) and seizure prediction horizon (SPH)

**Figure 3:**
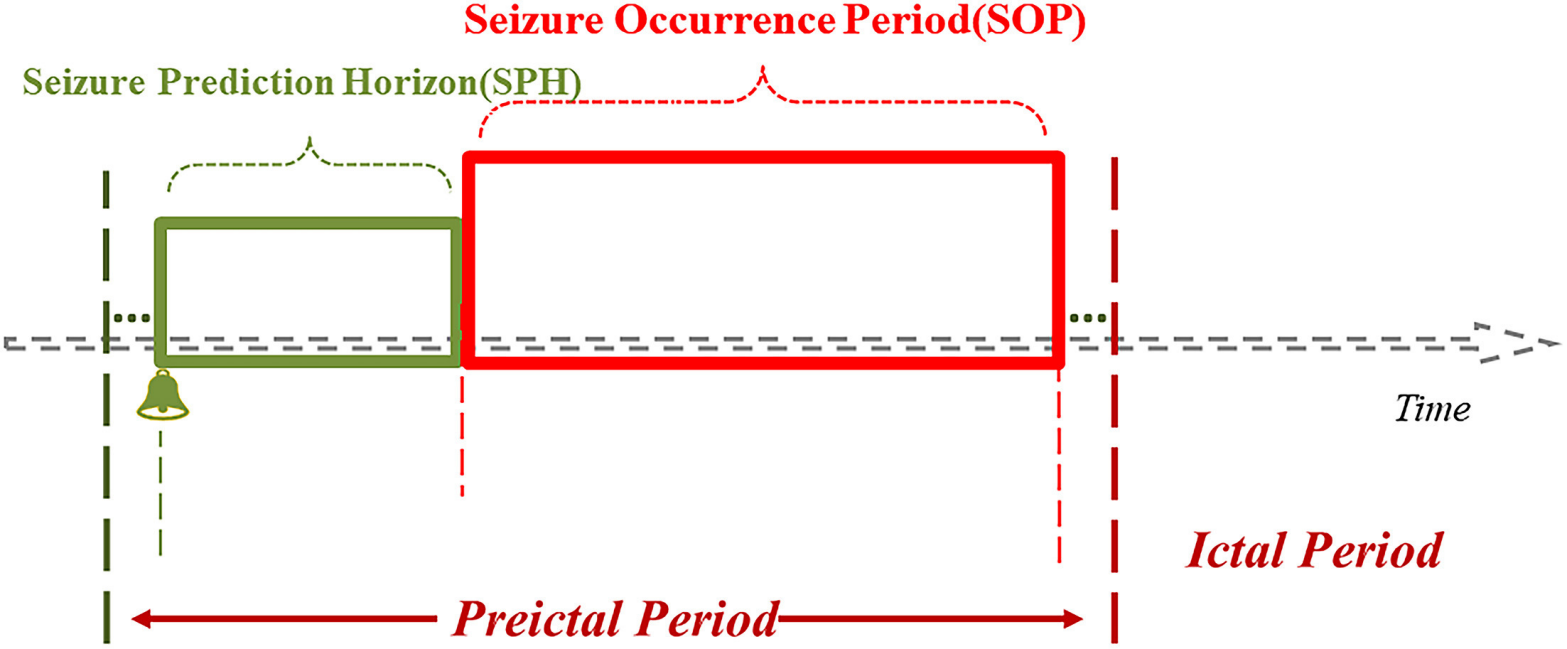
Diagramatic representation of SPH and SOP for seizure prediction.

In the event-based seizure prediction models, the preictal period (PP), SOP and SPH are predetermined, PP = SOP + SPH, and SOP and SPH are optimized for the model to achieve high sensitivity and low FPR (i.e., high specificity). There should be less uncertainty about the occurrence of the seizure after the end of SPH (i.e., ideally SOP = 0), and SPH should be long enough (83) so that measures can be taken by the patient and/or healthcare provider to prepare for or intervene and avert an upcoming seizure. The SPH refers to the period from the issue of the warning of an upcoming seizure to the beginning of SOP, within which no seizures occur; if seizures do occur within SPH, the model is considered to be underperforming. The preictal period (PP) is sometimes called the preictal interval length (PIL), the SPH the prediction period, and the SOP the prediction horizon (Figure 3) (71, 84, 85).

It has been suggested that the optimal SOP and SPH ranges to accurately predict seizures with high sensitivity and specificity should be distinct for each individual, that is, there should be no uniform, standardized SOP and SPH values across patients (86). In a conducted study, the best performance of a prediction model could be achieved when the SPH was <2 min (87), which is a low value and might be related to the algorithm used to construct the respective model as well as the quality of the raw data. SOP is defined as 30 min in the majority of the relevant literature, arguing that a 30 min SOP is appropriate as it is short enough so that it does not cause much anxiety to the patient then waiting for a seizure to occur after the end of SPH (33, 41, 42, 68, 71, 73, 88, 89). Zhang et al. (42) defined SOP as the 30 min and SPH as 0 min windows in practice and evaluated EEG records of 23 patients from a public dataset. The sensitivity of the shallow CNN model created to predict sEEG seizures was 92.2%, with the FPR of 0.12/h. Another study (73) reported the utilization of intracranial EEG (iEEG) data from 20 patients in a generalized dataset for the construction of a threshold-based model for predicting seizures. Here, the authors defined SOP as 30 min and 50 min and the SPH as 2 min. The final model achieved a mean sensitivity of 90.42 and 91.67%, and the FPR of 0.12 and 0.10/h, respectively. Another study that defined SOP as 30 and 50 min, respectively, used the Bayesian Linear Discriminant Analysis (BLDA) to construct models with sensitivities of 85.11 and 93.62%, and an average FPR of 0.08/h (41). The goal in constructing a good prediction model is to minimize SOP and maximize SPH, a combination that achieves the lowest possible FPR and the highest possible sensitivity.

### The shortcomings of current high-performance models

Although many of the predictive models reviewed in the current study have achieved excellent performance indices, there is still a great deal of uncertainty as to whether they can be used in the clinical setting. Firstly, most studies use the same public dataset, such as the CHB-MIT dataset or the Freiburg dataset, but there is a lack of validation of the constructed models in other datasets, which makes it difficult to guarantee that the same model will work well in different datasets. In addition the lack of available continuous datasets, which have to include multiple interictal, preictal, ictal, postictal EEG recordings, makes it difficult to obtain adequate validation of the constructed models. There are few eligible datasets, and only a few competitions have been conducted on the same datasets, the first one in 2002 at the First International Workshop on Seizure Prediction in Bonn, Germany (44). Not all datasets used in subsequent competitions were continuous over time. Again, the advanced algorithms that emerged from these competitions have rarely been validated on other datasets. In addition, most studies balance the interictal and preictal segments in order to obtain a high-performance model, but this is not true from a practical standpoint, since interictal periods are much longer than the preictal periods. For this reason, future studies should be validated on multiple continuous datasets without artificially balancing the number of segments over time or assuming constant preictal periods, SPH and SOP across seizures, even within the same patient. Adaptive individualized seizure prediction algorithms, such as the first one published one by Iasemidis et al. (18), should be capable of dealing with these shortcomings.

## Post-processing techniques in seizure prediction models

### Filter length, step size, and alarm threshold

**Figure 4:**
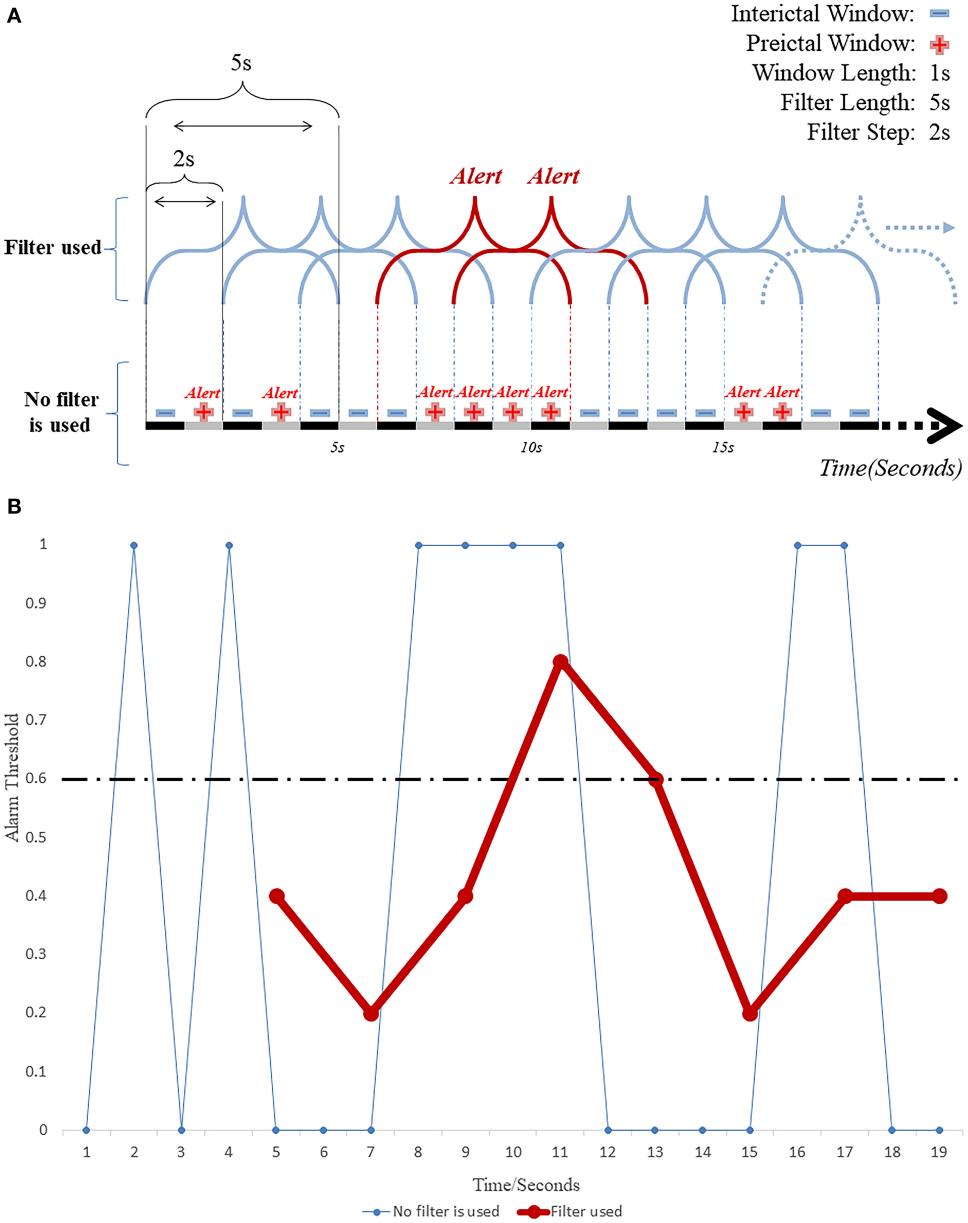
**(A)** Working diagram of EEG windows and the moving filter length and step size. **(B)** The repeated invalid early warning information is greatly reduced by filtering technology.

The post-processing techniques play important roles in the construction of the seizure prediction model as the interface between the “segment-based” model and the “event-based” prediction model. The post-processing methods help obtain the window segment outputs from the segment-based (classification) model (80). The most commonly used methods are filters, such as Kalman and Bayesian filters (35, 42, 54, 75, 77). Choosing the right filter length and alert threshold can improve the performance of the model to some extent. Determining the length of the moving filter is the first step in post-processing. The use of a filter can facilitate the elimination of most random errors from the classification model and reduce a large number of false predictions that may occur in the short term (Figure 4A). A study published in 2019 (55) used a causal moving average filter of 1 min in length to smooth and filter the classification results of 30 window segments, and the used 3D CNN (3D CNN) model obtained a sensitivity of 85.7%, and FPR of 0.096/h, and 10.5% of warning time ratio, as well. Another study (56) set the length of moving average filter to a range of 0–15 min, with a step size of 0.5 min, and found by comparison that the best performance of the prediction model was achieved with a moving average filter of 1 min in length, which is consistent with the findings of Ozcan et al. (55). Setting a very long filter length can enhance the smoothing of warnings resulting in delayed alerts or even missed seizures (low sensitivity), while too short a filter length can lead to opposite effects (high FPR and hence low specificity).

The alarm is the second step in the post-processing technique and it is ON when the detection rate of preictal segments within the pre-specified duration of the used filter reaches a set threshold (Figure 4B). Khan et al. (74) defined the probabilities of a segment in the classification model to be interictal, preictal or ictal as p0, p1 and p2, respectively, and assuming that p0 + p1 + p3 = 1. For monitoring and predicting seizures, the value of “1 – p0” was taken as the index of the model that would characterize a segment as preictal or ictal using a predetermined threshold of 0.6. When the probability exceeded 0.6, the model was programmed to issue an alert predicting an upcoming seizure. This setting was later verified by another group (56), using a threshold range of 0.1–0.9 in steps of 0.05. This study showed that the best performance of the prediction model could be achieved with a threshold value of 0.6, a sensitivity of 95.5% and FPR as low as 0.109/h. It was found that (57) the classification model constructed by combining CNN and features from a Directed Transfer Function (DTF) analysis was post-processed by a 20 point moving average filter reached sensitivity of 90.8% and FPR of 0.08/h. Theoretically, the predictive performance of the model can also be improved by estimating and not by having the threshold predetermined. For example, In the case of an artificial neural network (ANN)-based prediction model (80) constructed using a single nonlinear feature, the model generated alerts in the test dataset when the average eigenvalue in these periods was higher than or equal to those in the windows in the ictal period of the training dataset. The model achieved an average FPR of 0.014/h, with an average seizure prediction time of 26.73 min in advance of a seizure. These studies emphasize the importance of the employed thresholds since a very low threshold would end up sending too many false alarms, while an unnecessarily high threshold could lead to missing emergencies.

Although most false alarms can be eliminated in the post-processing stage, the ones that survive and repeatedly predict the same seizure will send out multiple warnings causing panic to patients (53). Hence, an “absolute refractory period (ARP)”, i.e., a period between two consecutive warning messages, should be set to prevent frequent warnings for the same seizure. The sensitivity and FPR of the model were 89.8% and 0.081/h, respectively, when the ARP was set to 20 min. In other studies, this ARP was equated to the SOP (55, 73), which improved the performance of the final model. Furthermore, the “absolute refractory period” setting can be useful in effectively removing incorrect warnings which escape the post-processing filtering stage. Ideally, only one correct warning before a seizure is sufficient for the necessary precautionary measures to be taken.

### Classification of EEG segments

**Figure 5:**
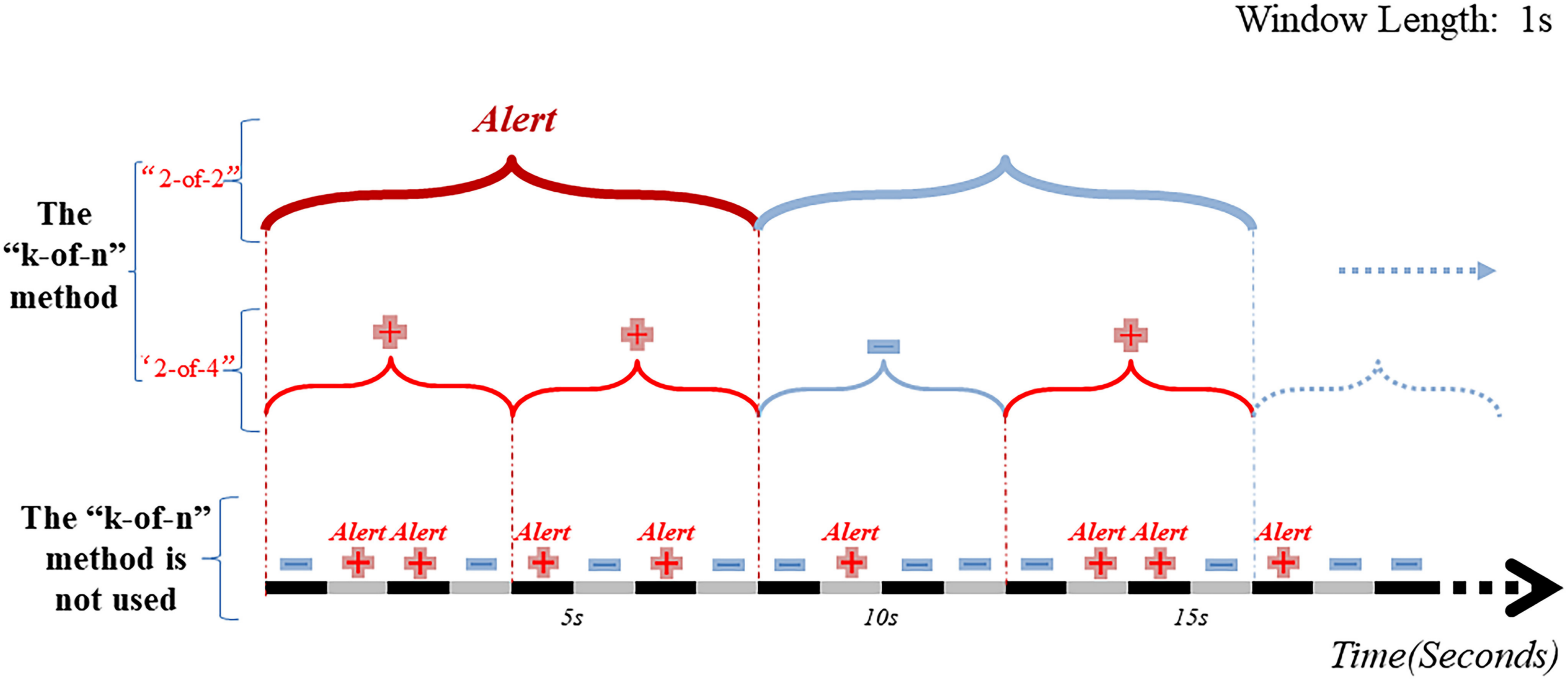
Schematic diagram of EEG window segment and post-processing hierarchy. Without using the “*k*-of-*n*” method, there will be 7 warning messages in the range of 0–16 sec. And after passing the two-layer “*k*-of-*n*” method, the model will send warning messages only at the 8th sec. The results show that the “*k*-of-*n*” method can greatly reduce invalid and overlapping warning messages.

The “*k*-of-*n*” approach means that if “≥*k*” of the consecutive “*n*” time window segments output from the classification (segment-based) model are determined “preictal”, then all “*n*” segments are considered preictal (Figure 5) (33, 36, 68). When the same post-processing method was applied to construct a CNN-based classification model for interictal and preictal periods, including only one layer of output regularization, the “8-of-10” was used to give an early warning. The proposed method exhibited better performance in all three datasets (Freiburg dataset, CHB-MIT dataset, American Epilepsy Society Seizure Prediction Challenge dataset) with sensitivities of 81.4, 81.2, and 75%, respectively (58). Parvez et al. (90) used an iEEG dataset of 27 patients to construct a binary classification model by Least-Squares Support-Vector Machine (LS-SVM) and defined the output of the classification model as “1” for the preictal window segments and “0” for the interictal window segments. Here, the authors compared different window lengths and numbers to derive the best combination. When 3 out of 5 consecutive 10-sec windows were identified as preictal, then all 50 sec were considered as preictal. If 2 out of 6 consecutive 50-sec windows were identified as preictal, then all 50 sec were considered as preictal. In case 2 out of 6 consecutive 50-sec windows were thus characterized preictal, an early warning would be issued. This prediction model was 91.95% accurate with an average of 2.14% prediction errors per patient. A subsequent study (36) used the same two levels of “*k*-of-*n*” (“3-of-5” and “2-of-6”), and the prediction model achieved the same excellent performance. The multi-level “*k*-of-*n*” method is superior to the single-level method.

Considering the uniqueness of EEG data from each epilepsy patient, studies suggested the importance of individualized seizure prediction models for precision medicine. So, the classification model has also been constructed without cross-validation from different patient datasets, thus ensuring a specific prediction model for each patient (91). In the post-processing steps, 3 levels of layer (window lengths of 5 sec, 2 and 6 min) of the “*k*-of-*n*” method were used for filtering. Each 2 min window that exceeded a certain percentage of 5 sec preictal or interictal windows was classified accordingly. When the characterized preictal 2 min windows within a 6 min window exceeded a different threshold, a warning of an impending seizure was issued. The average sensitivity of this optimized model was 94%, and the FPR was 0.111/h. These findings revealed that the “*k*-of-*n*” method was a simple and easy to implement post-processing technique. But the ideal “*k*” and “*n*” and the number of layers that optimized prediction were not estimated. The major drawback was that larger values of “*k*“ and “*n*“ would render the model less sensitive (delayed warnings or even missed alarms). Smaller values of “*k*” and “*n*” would impact the specificity of the model.

### Adaptive post-processing and firing power

In addition to the commonly used filter techniques and the “*k*-of-*n*” approach in post-processing, some studies have invoked novel post-processing techniques to further improve the performance of the epileptic seizure prediction model. A study (92) on real-time seizure prediction over a long period (169–364 days) used a new adaptive post-processing approach by updating the alarm threshold the alarm threshold every 7 days based on the prediction results of the previous week. The authors, considering the large number and overlapping outputs of the classification model, distinguished between preictal and interictal periods by comparing the mean and standard deviation of the Support Vector Machine (SVM) model outputs, which yielded a final model with a prediction sensitivity of 84%. Although SVM is one of the most commonly used traditional ML methods, a DL method can automatically extract features. But, if DL is to be used in seizure prediction models, the new methods need to be optimized and validated in the distinction of preictal and interictal periods. Direito et al. (22) emphasized the concept of Firing Power (93), first classifying windows from the test dataset into different periods through a classification model, then using this information to calculate the firing power parameters through a sliding window, and once the threshold threshold for Firing Power exceeded 0.5, the system generated an alarm of an upcoming seizure. So, new post-processing techniques are used today in the field of seizure prediction, but there is a lack of systematic validation of these algorithms on existing and new datasets.

## Effect of EEG data type and the employed AI method on the seizure prediction model's performance

### EEG data types and the model performance

In an “event-based” seizure prediction model, post-processing techniques and the length of SOPs and SPHs are important for the model performance. Also, we found that models built from invasive EEG (iEEG) recordings were generally superior to ones from scalp EEG (sEEG) recordings. Because iEEG has the advantages of less artifact and power interference, recording from brain sites closer to the epileptogenic focus, and without any attenuation from the skull, generates high quality of data with fewer confounding factors. A significant decrease in features important for the epileptic seizure detection and prediction algorithms was observed within 3 months after iEEG electrodes implantation. This may be due to trauma and tissue inflammation from the implantation. Also, the mean power of the high gamma band was found to decrease in many patients, which could inevitably affect the performance of the model (71). The sEEG is simple to perform, is non-invasive, standardized, and has fewer side effects, making it popular among epilepsy patients (72). Since sEEG is a non-invasive recording modality, it has significant disadvantages in terms of signal quality, signal artifacts, and lower spatial resolution of electrical brain activity. Peng et al. (94) used the same DL algorithm to construct seizure prediction models from sEEG and iEEG datasets. They showed the sensitivity of the prediction model constructed from the iEEG dataset exceeded that from the sEEG dataset by 5.8%, and the FPR was 0.08/h (lower than that of the sEEG model). The datasets used in most studies are mostly public, open-source datasets, such as the CHB-MIT dataset (physionet.org/content/chbmit) (95) (the most commonly used dataset for non-invasive EEG), and the Freiburg dataset for invasive EEG (which is now subject to a fee, and integrated into the European Epilepsy Dataset, www. fdm. uni-freiburg.de/EpilepsyData) (81); the American Epilepsy Society Seizure Prediction Challenge dataset, www.kaggle.com, and others (96, 97). These datasets have public and easily accessible characteristics, which facilitate the comparison of models' performance on the same EEG datasets, as well as support algorithms' optimization. The European Epilepsy Dataset (98) is the most enriched dataset available to date (www.epilepsiae.eu), including sEEG data from 225 patients and iEEG data from 50 patients to date. Moreover, it is essentially a combination of datasets from different recording sites, including Coimbra, Freiburg, Paris, and Treviso. However, since some of these integrated datasets are non-open access, it is difficult for most researchers to run their algorithms on these data. Importantly, most existing public datasets have a low number of cases and insufficient or discontinuous data. In the future, we should emphasize developing a large open-source platform to validate models and improve their performance while maintaining a good generalization capability.

The sampling frequency of the EEG may also affect the performance of seizure prediction models. For example, some studies have found high-frequency (80–500 Hz) oscillations (HFOs) to be a reliable biomarker for predicting seizures (82, 99). Since many commonly used public datasets contain EEG sampled at 256 Hz, HFOs cannot be detected. In addition, EEG recorded in long term [e.g., over days in the epilepsy monitoring unit (EMU)] is influenced by the patient's daily routine, the dose of anti-seizure medication etc. The above, in addition to the actual preictal period being very different and changing over time from one assumed by a model, constitute barriers for the use of predictive seizure models in practice.

### AI methods and the model performance

ML has been widely used to build seizure predictive models (100), where common algorithms such as SVM (101), random forests (102), and cluster analysis (32) are used. The principle of constructing predictive seizure models involves the pre-processing of interictal and preictal EEG datasets, followed by computational extraction of commonly used features, and then constructing classification models by ML. In a “segment-based” epileptic seizure prediction model (70), the sensitivity reached was 95.8% using SVM. DL is derived from ML, and the biggest difference between DL and ML is the ability of DL to better mine data features (29). Automatic feature extraction means less human error, and the underlying mathematical logic of DL algorithms is more rigorous and suitable for processing and handling big datasets. The performance of the classification model constructed by the algorithm determines, to some extent, the performance of the epilepsy prediction model processed by post-processing techniques. In general, models constructed by DL outperform ML, and ML often outperforms DL in terms of its ability to perform on brand new data (generalization capability/robustness).

## The influence of features on prediction of seizures

Seizure prediction systems focus more on the patterns (features) of EEG changes over short cycles, hours or minutes. Some recent studies have reported that circadian rhythms may affect cyclical seizure occurrences over days or months, which is likely to be related to the patient's hormone levels and psychological factors (103). For example, in clinical practice, there are cases of women of childbearing age who experience regular menstrual seizures, and patients undergoing seizures when they are stressed, emotionally distraught or tired (104). Public EEG datasets over short periods of time do not capture these physiological factors. Preictal critical slowing down as a feature in the EEG over hours to days is hypothesized to also have predictive value of seizures occurrence (105). In a study on predicting drug responsiveness in epilepsy patients (106), an SVM model constructed by extracting features of brain network connectivity obtained a sensitivity of 94%. The feature of effective connectivity in the frequency domain has constituted the basis for development of novel biomarkers for epileptogenic focus localization from interictal periods (107, 108) as well as for evaluation of the risk to status epilepticus (SE) and sudden unexpected death in epilepsy (SUDEP) (109, 110). Availability of more biomarkers to predict seizures should provide theoretical and technical support for more accurate seizure prediction. Previous AI studies have ignored such physiological factors as well as the use of newly discovered features, which may explain the difficulty of successfully applying high-performance models in the clinical setting.

## An EEG-based model for predicting and detecting seizures

Seizure prediction's goal is to detect the preictal period while seizure detection's goal is to detect the seizure's onset. Seizure detection could be considered as a subset of the seizure prediction problem (e.g. when SOP is zero with a specified SPH) (3, 70, 111, 112). In a detection model constructed using the cross bispectrum features of EEG signals to identify preictal windows (70), 75 of 78 seizures were eventually successfully detected. However, predicting seizures is more advantageous than detecting seizures and is more relevant to the needs of epilepsy patients (113). Predicting seizures can alert patients in advance so that they can take or inject medication or stop current risky activities to avoid seizures or mitigate seizure hazards.

## Recent research developments

It has been for a while since researchers sought to promote the practical application of high-performance models for predicting seizures including individualized and adaptive models per patient (18, 20, 114, 115). Along similar lines of considering the variability across patients, Yang et al. (116) have recently proposed a Self-Supervised Learning ML system for predicting seizures, training a prediction model based on changes in EEG characteristics unique to each patient before a seizure resulting to a more robust performance of this adaptive model. In addition, some researchers have focused on other than EEG physiological changes in patients, such as hormonal levels, mood changes, circadian rhythms, as well as on different types of epilepsy, which could lead to more accurate seizure prediction (62). NeuroVista (45, 63) implanted epilepsy monitoring systems in 15 epilepsy patients, allowing for the first time long-term (over months) monitoring and recording (segmental and only from 3 patients with 2 years follow-up) of their EEG. Such databases are important to have for the development and validation for epilepsy prediction models (46). The closed-loop responsive neural stimulation (RNS) system developed by NeuroPace has been implanted to patients to provide stimulation when abnormal EEG patterns changes are detected (47). Again, a question here is how much of the recorded EEG from such devices is stored in a database for future applications. A study that followed patients for 45 months after implantation of the RNS system reported improvement of seizure control over time (years). It also noted that implantation of the device was demanding on the physician and the equipment, and that there was a risk of infection and brain injury to the patient (48). In this sense, sEEG seizure prediction systems, together with non-invasive electrical or magnetic stimulation devices, may be more applicable to clinical practice in the future but are currently less well-studied.

## Future perspective

Because of the “random” (probabilistic view of the) or “chaotic” (deterministic view of the) nature of seizure occurrences, there is an urgent need for new technologies that could precisely predict seizures in advance, which could then ideally drive timely intervention schemes prior to seizure occurrences. Along this line, the construction of high-performance seizure prediction models using advanced computer technology and AI has received increasing international attention. However, the field of predicting seizures still faces several difficulties and shortcomings that affect the performance of the current seizure prediction models and constitute the most critical obstacles for their translational route to clinical practice.

### EEG data

a. Importance of large open-source databases and exchangeable platforms: Currently, there are no big open-source datasets available to validate prediction algorithms, while both ML and DL rely on the support of big data. Although many current models have achieved high performances with pre-determined parameter values and assumptions on particular datasets, they would perform poorly in clinical practice when these assumptions and values are not valid. It is expected that good communication would allow validation of internationally advanced ideas as well as techniques and inspiration from the GitHub platform in computing.

b. Refinement of clinical characteristics of the data: It is well known that differences in age, gender, medication status, seizure type, and other patient characteristics and demographics, which leads to a high number of confounding factors in the raw data, thereby affecting the performance of models to varying degrees and hindering their real-life application. The current EEG datasets still lack the necessary inclusion and exclusion criteria to compensate for the above, probably due to less integration of action between clinicians and model developers.

c. Addressing the shortcomings of the low signal-to-noise ratio of sEEG data: Noninvasive BCI technology using sEEG has unique advantages. sEEG is safe, simple, convenient, and universal, which makes it easier to record from more patients under different conditions. Importantly, collaboration between engineers and computer scientists has led to development of pre-processing techniques for sEEG that eliminate interference artifacts with high fidelity.

### Algorithms and models

a. Disclosure and availability of algorithms: The vast majority of research studies are currently focusing on the innovation, upgrade and validation of new algorithms, and comparison of old and new seizure prediction models on the same dataset. Establishing an open-source platform that allows validation and modification of models using different datasets would improve the generalization (robustness) of models and help address key issues of model-to-clinic transition.

b. The choice between ML and DL: ML requires manual feature extraction, which is prone to feature mismatch. While DL needs no manual feedback for feature extraction, there is a problem of over-extraction of features, which leads to weak generalization of the resulting model. It is believed that ML should be combined with DL, that is, to extract EEG features by DL and build classification models using traditional ML methods, thus obtaining generalizable and high-performance models.

### Post-processing of results

Post-processing techniques play an important role in bridging the (segment-based) classification and the (event-based) prediction models with a significant impact on their performance in seizure prediction. Currently, researchers in the field of EEG-based seizure prediction has not paid enough attention to post-processing techniques. Although multiple methods and techniques have emerged, there is no metric to evaluate the advantages and disadvantages of these techniques. In the future, relevant evaluation metrics should be developed to evaluate post-processing techniques.

### Metrics of performance

a. Sensitivity and false prediction rate: Sen and FPR are important metrics of performance of prediction models in clinical practice.

b. SOP and SPH: The SOP and SPH are pre-determined parameters that affect Sen and FPR of any prediction model. The longer the SPH and shorter the SOP, a better seizure prediction model is constructed. In today's precision medicine paradigm, the values of SOP and SPH could be optimized to get the best Sen and FPR for an individual or class of individuals.

### Prospective studies

Many high-performance algorithms have been developed and tested on collected data retrospectively. It is thus difficult to determine whether they generalize well. A small number of epilepsy patients have been recruited for prospective analysis by companies and teams, but overall progress remains limited. Future validation on multiple datasets, which could later be replicated in a number of competitions, is highly recommended.

The application of models to clinical practice should also consider certain practical issues, such as running in real time, low power consumption for implantation in the brain or embodiment in wearable devices, user-friendliness of inputs and outputs to and from the models.

## Conclusion

Classification empowered by ML and DL constitutes a basic component of seizure prediction models. Nevertheless, without post-processing techniques and proper (a priori) determination of SOP and SPH, there exists no high-performance model that predicts seizures. Hormone levels, psychological factors, medication and blood levels of antiepileptic drugs can all influence the accuracy of predictive seizure models. In the future, more consideration needs to be given to these factors that are characteristic per epilepsy patient as their combination with the EEG features could facilitate the development of better and applicable to clinical practice seizure prediction models.

## Author contributions

ZR primarily did the literature review and wrote first draft. ZR, XH, and BW participated in the discussion and drawing. XH obtained funding. All authors contributed to the article and approved the submitted version.

## Funding

This work was sponsored by Henan Province's Gong Jian Program (Authorization number: SB201901074), 23456 Talent Engineering (Authorization number: ZC20200371).

## Conflict of interest

The authors declare that the research was conducted in the absence of any commercial or financial relationships that could be construed as a potential conflict of interest.

## Publisher's note

All claims expressed in this article are solely those of the authors and do not necessarily represent those of their affiliated organizations, or those of the publisher, the editors and the reviewers. Any product that may be evaluated in this article, or claim that may be made by its manufacturer, is not guaranteed or endorsed by the publisher.
